# Open Science is for Aging Research, Too

**DOI:** 10.1093/geroni/igz028

**Published:** 2019-09-04

**Authors:** Derek M Isaacowitz, Majse Lind

**Affiliations:** Department of Psychology, Northeastern University, Boston, Massachusetts

**Keywords:** Methodology, Quantitative research methods, Research methods

## Abstract

In response to concerns about the replicability of published research, some disciplines have used open science practices to try to enhance the credibility of published findings. Gerontology has been slow to embrace these changes. We argue that open science is important for aging research, both to reduce questionable research practices that may also be prevalent in the field (such as too many reported significant age differences in the literature, underpowered studies, hypothesizing after the results are known, and lack of belief updating when findings do not support theories), as well as to make research in the field more transparent overall. To ensure the credibility of gerontology research moving forward, we suggest concrete ways to incorporate open science into gerontology research: for example, by using available preregistration templates adaptable to a variety of study designs typical for aging research (even secondary analyses of existing data). Larger sample sizes may be achieved by many-lab collaborations. Though using open science practices may make some aspects of gerontology research more challenging, we believe that gerontology needs open science to ensure credibility now and in the future.

**Translational Significance**

Open science practices will help ensure that our translational efforts are based on solid, credible science.

Although some areas of psychology, biology, and medicine have embraced open science principles, the field of gerontology has been slower to modify its practices to reflect open science. In this article, we consider why aging researchers should care about open science, challenge some of the roadblocks often cited by aging researchers for not adopting these practices, and offer concrete steps that we believe are feasible for many (if not most) researchers who study aging to implement to push their research, and our field, in the direction of open science.

Open science refers to “the practice of science in such a way that others can collaborate and contribute, where research data, lab notes and other research processes are freely available, under terms that enable reuse, redistribution and reproduction of the research and its underlying data and methods” (https://www.fosteropenscience.eu/foster-taxonomy/open-science-definition). Increasing availability of research and research products through open access may broaden interest in research and widen the ability of research to affect people’s lives. Open science practices can increase educational impact from university-based research to the general public (Howe, Howe, Kaleita, & Raman, 2017). As open science offers a platform in which research methods and codes can be more easily and widely shared among researchers, collaborations can form more quickly, and new tools can be applied more rapidly to new data sets and new problems. Open research is also associated with more citations, increased media coverage, and better funding opportunities (McKiernan *et al.*, 2016); this may also hasten translational impact of new research as well. Open science practices are also critical to make the outputs of scientific research (eg, publications) more credible (Spellman, Gilbert, & Corker, 2017). Our article focuses on why gerontology in particular needs open science to increase credibility.

## Why Do We Need Open Science?

The move toward open science not only grew out of its many benefits but also from questions about the integrity and trustworthiness of academic science. One set of concerns involved the lack of transparency in research. Notably, researchers aiming to gain a deeper understanding of other researchers’ work were often unable to gain access to the material and data (Spellman *et al.*, 2017). Sometimes requests would never be answered, or research material and codes were not available because they were stored inadequately and eventually lost. Furthermore, many studies were never published, instead lost to the “file-drawer” because the results were not as hypothesized and/or did not reach statistical significance (Fanelli, 2012). Certain research practices revealed to have been commonplace though not disclosed in publications—such as leaving out outliers without a valid reason to do so and/or not mentioning conditions that did not “work”—also led to concern about the robustness of the published literature (Martinson, Anderson, & de Vries, 2005). Unfortunately, though rare, cases of scientific fraud have also driven the need for a more transparent and thus trustworthy scientific practice. Prominent researchers in several fields have been accused and convicted of research fraud.

Beyond concerns about the lack of transparency in the scientific literature, failure to replicate previous published findings has been another strong motivator for promoting open science (Bohannon, 2013; Spellman *et al.*, 2017). For example, in the psychology field, alarming results came from multiple labs combining resources to conduct highly powered replications of previous studies. Although results showed that the majority of classic effects were replicated (Klein *et al.*, 2014), a follow-up project including both classic and contemporary effects revealed that only 3 of 10 studies replicated (Ebersole *et al.*, 2016), and several other pre-approved, large-scale replications (Registered replication reports) showed similar results (Simons, Holcombe, & Spellman, 2014). In the Reproducibility Project: Psychology (Open Science Collaboration, 2015), more than 270 researchers engaged in replicating studies from articles published in 2008 in top journals. Similar to the previous projects, whereas 97 of the original findings were statistically significant, only 35 of the replicated studies were statistically significant, and less than half of the replications revealed effect sizes that fell within the 95% confidence interval of the original studies.

In the field of economics, Chang and Li (2015) aimed to replicate 67 economic articles published in 12 high-impact journals using author-provided data and codes. Aside from six articles that use confidential data, they obtained data and code replication files for 29 of 35 articles (83%) that were required to provide such files as a condition of publication, compared with 11 of 26 articles (42%) that were not required to provide data and code replication files. The authors successfully replicated the most important qualitative result of 22 of 67 articles (33%) without contacting the authors and 29 of 59 articles (49%) were replicated with assistance from the authors. With less than half of the articles successfully replicated, the authors emphasize that replication concerns also exist within the field of economics. Because most of the replication failures across disciplines involve significant effects that fail to replicate, it would appear that false-positive findings are quite prevalent in published research (Simmons, Nelson, Simonsohn, 2011).

The Reproducibility Project: Cancer Biology (RP:CP)—a collaboration between the Center of Open Science and Science Exchange—aimed to replicate experiments from high-impact journals published between 2010 and 2012 in the area of cancer biology (Errington *et al.*, 2014). For each article, a registered report detailing the proposed experimental design and protocols for the replication was peer-reviewed and published before data collection, and the results were later published as a Replication Study. The first 10 of 18 studies were published in *eLife:* Two of the studies failed to replicate, two were inconclusive, and six were partly reproduced, resulting in a replication percentage around 40%. The second round of results, also published in *eLife*, was more encouraging and showed that important parts of 4 of 5 replication studies were replicable. Eventually, the project had to be scaled down and then discontinued because reproducing this amount of studies was time-consuming, expensive and largely inconclusive. The point of course was not to try to directly replicate every finding in the literature, but rather to estimate the replicability of prior studies in general.

As a reaction to the concerns about reproducibility in biomedical research, the Federation of American Societies for Experimental Biology (FASEB) invited experts from its member societies (including GSA), representatives from the National Institute of Health (NIH), National Science Foundation (NSF), and other stakeholders to discuss ideas for improving reproducibility and transparency in biomedical and biological research—ideas that were later published as a report holding 15 recommendations (Antin *et al.*, 2016).

Gerontology subsumes a range of disciplines, from social and behavioral to biomedical sciences. In response to replication failures across these disciplines relevant to gerontology, the editor of the interdisciplinary GSA journal *The Gerontologist* (Pruchno *et al.*, 2015) admitted that they, along with many other journals, have been focusing too much on promoting new and innovative research and not enough on replication studies, and announced that they would start encouraging authors to also submit high-quality replication research. However, we are not aware of any specific replication studies that have been published in *The Gerontologist* to date, and when investigating the author guidelines of prominent journals in aging, including *The Gerontologist*, only *the Journal of Gerontology Series B* mentions replication studies explicitly.

## What Practices Contribute to Low Replicability?

In the search for reasons why so many past findings across areas of academic science do not replicate, a number of questionable research practices (QRPs: John, Loewenstein, Prelec, 2012) were identified as likely contributors to the false positives appearing in the literature. As such, issues of transparency and replicability are interrelated, as lack of transparency may increase the use of QRPs, and more transparency is expected to reduce their use.

In terms of data collection, the field may suffer from a *confirmation bias*—the search for evidence that confirms preexisting beliefs or theories while either ignoring or downplaying evidence inconsistent with expectations. An example of such practice is *optional stopping,* in which researchers stop collecting more data when they find results that confirm their expectations (Spellman *et al.*, 2017). This increases the risk of false-positive results because this group of participants may differ from the final power-estimated sample of participants. This may explain in part why later researchers, perhaps not as attached to the same expected findings, fail to replicate the findings in future attempts.

Of course, the concern may also be raised that replicators may have their own agenda (such as hoping to disconfirm the previous findings: Bissel, 2013). There are concerns that how failing to replicate prior published research can be seen as doing damage to the original authors’ reputation (though replicators are often careful to not hold original authors’ personally responsible for QRPs that were widespread previously). We do not think that replicators have *more* responsibility to engage in good practice than the original authors do. Both original authors and replicators should be held to the same rigorous standards. That said, replicators may benefit from direct, open communication with the original authors of the work they want to replicate. This speaks to the importance of research transparency even in the context of replication.

With respect to data analysis and reporting of results, the field may suffer from *Hypothesizing After the Results are Known* (*HARKing*: Kerr, 1998) or post hoc *explanations*—collecting data and then interpreting it retrospectively, as though what was found had been expected in the first place (even though it was not). Nosek and colleagues (2018) emphasize the importance of distinguishing between predictions and post hoc explanations (*postdictions)*. Interpreting postdictions as predictions can lead to overconfidence in vague findings and inflate the likelihood of believing that there is evidence for a finding when there is not. Presenting postdictions as predictions can falsely reduce uncertainty and ultimately decrease reproducibility. This is problematic because outcomes appear as more predictable after they have been observed, a tendency called *hindsight bias* or the “I knew it all along” tendency (Spellman *et al.*, 2017). When researchers find something in their data and act as though they predicted in when in fact they did not, they encourage the reader to falsely inflate their belief in the robustness of the findings. In our opinion, researchers should have more confidence in hypothesized results than surprising unhypothesized ones, though both should be publishable when appropriately labeled as such.

*P-hacking* is another QRP, in which *p*-values are misused to get a significant finding at the < .05 level. For example, data might be rounded or analyzed in multiple ways, such as including covariates, until significant results are reached. Historically non-significant findings have been much harder to publish than statistically significant findings; this ongoing pressure may partially drive many of the QRPs including *p*-hacking. Statistics reveal that 96% articles in psychology show significant effects (Bakker & Wicherts, 2011) and 80% on average across sciences (Fanelli, 2010). Relatedly, only measurements supporting preferred theories and findings may be published, resulting in a *measurement bias*. Taken together, these practices contribute to a *publication bias* explaining why many results do not replicate later on, because use of these QRPs leads to a literature filled with false-positive findings.

After a number of years focusing on why the prior literature may not be as credible as had been hoped, interest has shifted to ensuring that research practices moving forward are more likely to produce credible findings. A growing number of quantitative (Nosek *et al.*, 2015) and qualitative (Haven & Van Grootel, 2019) researchers are working on implementing ways to use open science in their research to boost the credibility of academic science and to reduce QRPs.

## How Can Open Science Practices Reduce the Use of QRPs?

The main goal of open science is to increase transparency, reduce QRPs, and overall to increase the credibility of published research findings. Preregistration is a key component of open science in which details regarding data collection methods, analysis plans, and rules for data exclusion can be registered in advance (https://osf.io/prereg/). Preregistering study designs and analyses document the specific number of participants required to reach adequate statistical power to detect meaningful effects, thus requiring researchers to conduct a priori power analyses to determine sufficient sample sizes for hypothesis testing. Thus, from an early stage, authors will have to think carefully about study designs that match the available participants and resources. Research fields like clinical psychology (Tackett, Brandes, & Reardon, 2019) have suffered from sample sizes too small to detect small and medium effect sizes, and poor statistical power has been a problem in psychology in general (Fraley & Vazire, 2014). This may be a particular concern in disciplines defined by investigating group differences. In other research fields, samples might be too big and overpowered. Open science practices involve awareness of how to estimate the necessary sample sizes to answer particular research questions, and also getting researchers to commit to these justified sample sizes before starting a study. This should decrease false-positive (and also false-negative) results. In addition, preregistered sample sizes should prevent researchers from *optional stopping*, resulting in more reliable effect sizes.

In data analysis and processing, preregistration will also minimize *HARKing* because preregistered hypotheses and analyses can be compared with the published article, providing a clearer picture of whether the initial hypotheses were supported or not (Haven & Van Grootel, 2019; Miguel *et al.*, 2014; Nosek *et al.*, 2018). Preregistering hypotheses and analyses mean that the researcher has clearly stated what pattern of results would support what theories in advance, and why. It also means that an analysis plan has been laid out in advance of seeing the data. Data-driven exploratory analyses can generate interesting tentative findings that can be confirmed in follow-up studies. Preregistering a study does not make it impossible to do additional data-driven exploratory analyses; it simply makes it easier to clearly discern which analyses are confirmatory and which are not. Thus, the *confirmation bias* will be reduced as attempts to only report results supporting existing theories will no longer be an option. *Hindsight bias* (“I knew it all along”) will no longer be a temptation because researchers will base their reporting on the initial ideas and measurement bias will also be eliminated because it will not be possible to simply ignore certain measures. These changes may actually free researchers from the pressure of having to try to ensure that their results align with their hypotheses; in this way, preregistration may actually serve to relax pressure that may increase the risk of QRPs.

Registered reports are another open science initiative to both reduce HARKing, *p*-hacking, and other QRPs that can lead to spurious statistically significant findings, as well as to minimize the publication bias that arises from journals’ tendency to only publish articles featuring statistically significant results. Registered reports are empirical articles that undergo peer review at the initial design phase of the project. Following peer review of the study background and methods, authors receive an “in-principle” acceptance from the journal, meaning that as soon as the study is completed it will be published in that journal regardless of the outcome (as long as the planned methods are followed). That is, publication decisions do not depend on finding significant results, but on the quality of the methodology and analysis, allowing questions to be tested in a more impartial way. Whereas deviations from preregistration in a usual article will just need to be explained in the submitted manuscript, deviations from the research plan in an accepted registered report would need approval from the editor. Although conducting and publishing research using the registered report format takes more time for researchers during study planning, and more involvement from editors before the study is conducted, it may help both researchers and journals increase research quality and transparency.

Preregistration and registered reports are not the only tools of open science. Other open science practices, such as making data, materials, and code publicly available, can also reveal *p*-hacking, allow verification from third-parties, reduce mistakes, and pave the way for a more realistic proportion of significant and non-significant findings in the published literature. Providing data and code availability contributes to a more honest science, and for some researchers increased transparency may be their main pivot toward open science, but it is important to keep in mind that research transparency alone is not enough for a more credible science overall. Dishonest reporting can be misleading, but simple honest transparency is not sufficient for research credibility: for example, transparent reporting cannot make an underpowered study adequately powered, or make an inappropriate analysis appropriate (see discussion in Gelman (2017)). Therefore, higher transparency and better research practices are *both* necessary strategies for a more credible science.

Taken together, open science practices will reduce several QRPs and thereby decrease the *publication bias* in academia, thus reducing the prevalence of false positives in the published literature. In addition, embracing equivalence testing (Lakens, McLatchie, Isager, Scheel, & Dienes, 2018) to aid in interpretation of potentially meaningful null effects (such as between-groups tests of age differences) will reduce publication bias and the impulse to *p*-hack by allowing more null effects to be published (see also Isaacowitz (2018)). Taken as a whole, these practices should lead to a more credible, and accurate, scientific literature.

We see open science policies as an important step towards also improving the research practice in gerontology. Some small steps toward open science practices have already been initiated. For example, some gerontology journals (eg, *Aging Cell, Neurobiology of Aging, Psychology and Aging*) encourage or—in only one case—even require (eg, *Aging*) authors to make their data publicly available. However, a recent study revealed that even high-impact journals with good intentions of open science (eg, Science, which requests code and data availability) failed to implement these practices (Stodden, Seiler, & Ma, 2018). Of 204 randomly selected studies, artifacts from only 44% could be obtained, and only 26% could be reproduced. Thus, without concrete and field-wide efforts, it is likely that the gerontology journals also fail to follow-up on their good intentions of open science. Later, we discuss in more detail why the field of gerontology specifically would benefit from integrating open science practices.

## Why Does Aging in Particular Need Open Science?

We believe that aging research is at least as susceptible—perhaps even more so—to a literature rife with false-positive results, making open science, especially relevant for our field. Until recently, almost every published aging study concerned statistically significant age differences in some measure, be it a behavior or a psychological, biological or sociological process. Given the ambiguity associated with non-statistically significant findings regarding age differences, and the purported lack of conceptual relevance of such findings when most aging theories focus on documenting and understanding age differences, the fact that almost every study in the literature presents significant age differences on one or more variables may reflect a publication bias such that only the studies showing age differences have been published (Isaacowitz, 2018). Although we are not aware of any specific studies that would provide empirical support for this, it seems logical: Until recently, null age effect studies were largely not publishable and thus would have likely ended up in the file drawer. This practice would inflate the estimate of age differences throughout the published literature.

QRPs in gerontology may further lead to an even more unreplicable and uncredible literature, more biased with false positives than might occur from publication bias alone. Given the strong incentives to report significant age differences, researchers may search for relationships that reach statistical significance and act as though these were the relationships they were expecting in the first place (HARKing). They may look at the data after running groups of subjects and stop collecting data when the groups are significantly different from each other (optional stopping). If an analysis is close to the p < .05 level of significance but does not land below that threshold, researchers may add covariates, remove outliers and the like to try to get the effect below the .05 significance level (*p*-hacking). Each of these practices will make false-positive findings more likely and will render the literature unreliable with vastly overstated estimates of the magnitude of any age differences.

Simply put, the use of any or all of the described QRPs in aging research renders our accumulated scientific literature less trustworthy. One survey of psychology researchers found that 31% of developmental researchers admitted to engaging in QRPs (cf. 27% in clinical and 40% in social: John *et al.*, 2012). However, it does not need to be shown that the prevalence of such QRPs is any greater in aging than in other subdisciplines for QRP to be a real concern for aging research. Given that most of our conclusions in the aging literature are based on finding significant group differences on some psychological (or other) measures as functions of age-group membership, the use of QRPs in a subset of the literature, combined with the effects of publication bias in favor of positive findings, suggests that the nature of observed age differences in the published literature may be inflated. Some major course correction is thus warranted.

One additional concern in the aging literature, at least in the subfields we are most familiar with, is that studies are rarely designed to test competing hypotheses. Rather, the tendency is to design studies to support a single conceptual framework. This is not problematic by itself; rather, the concern happens in such studies when only findings that support given theories are used to update beliefs in those theories. In contrast, null or contradictory findings are not symmetrically used to update beliefs away from those theories. Hypothesis testing that can only be used to support prior beliefs but not to modify them leads to scientific literature that inflates the perceived support for existing theories if published findings are not used to reduce support for them (Ledgerwood, 2018). This is one reason that preregistration needs to include not only analyses, but clear (falsifiable) predictions as well, so that findings are used either to increase or to decrease belief in particular theories.

In summary, we believe that the gerontology literature likely is replete with false positives, especially concerning age differences that may in part be the result of (conscious or unconscious) QRPs that have been typically used in research but are now realized to be problematic practices. We can acknowledge that the literature to date may be less credible than hoped as a function of these past practices while focusing our energy instead on ensuring better practices moving ahead. In that spirit, we now turn to describe open science efforts that may be especially relevant and productive for gerontology. We will also consider challenges and obstacles for implementing such practices, in the hope that an open discussion of possible roadblocks will help the field push to a more open credible science. Note that research credibility can only be achieved by incorporating multiple elements such as (a) transparency (b) preregistration, (c) replication, and (d) higher standards for the quality and quantity of evidence needed to make strong scientific claims (Vazire, 2018).

## What Open Science Practices Are Most Relevant to Aging?

We believe that a variety of open science practices will be useful to gerontology researchers, and can increase the credibility of our published research. The Open Science Framework (osf.io) is one online clearinghouse for open science practices. On OSF, researchers can post materials, data, and analysis code, and record study preregistrations. Although OSF may be the best-known online resource, there are many other online services that also support preregistration, such as ClinicalTrials.gov, AEA Registry, EGAP, Uri Simonsohn’s AsPredicted, and trial registries in the WHO Registry Network. There are also numerous online data repositories, such as Dataverse, Dryad, and Figshare.

We view the increased use of open data and open methods as well as preregistration as a clear path for gerontologists to adopt a critical open science practice. We also understand that researchers may have reservations about these methods, or may feel that they do not apply to their research. Thus, we consider types of preregistration and open data options in more detail, to illustrate how they are widely applicable for different types of aging research.

A search on preregistered “aging” studies in the OSF database revealed a total of 254 preregistered studies from 2014 to 2019 (search date: March 26, 2019). The number of preregistered aging studies has increased in this 6-year period: 5 studies in 2014, 14 in 2015, 20 in 2016 (three of them withdrawn), 74 in 2017 (one withdrawn), 114 in 2018 (one withdrawn), and so far 27 in 2019. Note that this search only encompassed studies with “aging” in the research title and the accurate number of aging studies may therefore exceed 254. However, the preregistered studies and studies using open data and methods unquestionably make up a small amount of all published aging studies.

OSF and open science proponents have worked hard to develop eight official templates (plus one working draft) that allow researchers to commit in advance to analytic decisions (such as what will happen if models do not converge) and to distinguish confirmatory from exploratory analyses, thus helping readers calibrate their beliefs (about the truth of conceptual models, for example) specifically according to the reported results. Of course, deviations from the preregistration can later be explained and justified; the important point is that the strength of the evidence presented in the final published article should be weighted according to the closeness to the preregistered analyses and hypotheses, so exploratory findings should be weighted less strongly (Vazire, 2017b).

These templates provide aging researchers with opportunity to adopt a key open science practice in ways that are suited for the type of research they conduct, In Table 1, we list the eight official templates (and one working draft) and elaborate on their relevance for aging research. Although the templates in the table focus on preregistration, OSF is also suitable for other open data and open methods purposes. For example, OSF can be used to create project pages; there, researchers can upload study materials, code, and data. Recent guidelines (Mellor, Vazire, & Lindsay, 2018) suggest that codes posted to OSF should be executable (including information about what software and version is needed, any settings that should be used, etc.) and should be sufficient to reproduce all the results presented in the manuscript. OSF can also be used to upload underlying descriptions of the method and protocols for others to gain a thorough understanding of the research study. The studies in OSF are not monolithic: they range widely from highly detailed and thought-out studies with detailed preregistrations to less detailed studies without clear hypotheses. We encourage all aging researchers to start including open data/open method and preregistration as part of their research routines, making use of the resources on OSF, and to register the most amounts of study details possible: Something is better than nothing.

**Table 1.** Open Science Templates From Open Science Framework (OSF; osf.io) Relevant for Aging Research

Template name	Template information	Relevance for aging
“The OSF Preregistration” (https://osf.io/zab38/wiki/home/?view)	This is the most frequently used template, so far, and consists of a series of research questions for *before* data collection. This template can also be used for exploratory research as long as the hypotheses are clearly stated as exploratory.	The template offers documentation of classic study elements and thus fits a wide range of study designs in the aging field.
“The Open-Ended Registration” (https://osf.io/haadc/)	This template serves as a time-stamped snapshot of a research project.	The template is useful when aging researchers wish to register a completed project.
“The AsPredicted Registration” (https://osf.io/fnsb6/)	This template consists of eight research questions obtained from the content recommended by AsPredicted.org.	The template offers a series of fundamental research questions that apply to a variety of aging studies.
“The OSF-Standard Pre-Data Collection Registration” (https://osf.io/9j6d7/)	This template is useful *after* the data collection has begun and consists of a series of questions about the data collection.	Aging researchers can use the template when the pre-analysis plan is already uploaded on OSF.
“The Replication Recipe: Preregistration” (https://osf.io/4jd46/)	This template applies to preregistration of replication studies and includes a series of questions about the original study and the replication study.	We encourage aging researchers to conduct replication studies using this template as documentation.
“The Replication Recipe: Post-registration” (https://osf.io/9rp6j/)	This template can be used to register a replication study *after* the study has been conducted.	Should aging researchers already have completed the replication study, they can then register the study using this format.
“Preregistration in Social Psychology: Preregistration” (https://osf.io/ce3hr/)	This template can be used to register a research study in social psychology outlining the hypotheses, methods, and analysis plan.	Aging research with aspects from social psychology (eg, social roles and prejudices across the lifespan) may find this template useful.
“Registered Report Protocol Preregistration” (https://osf.io/gm36s/)	This template is useful to register a research protocol *after* having been given “in-principle acceptance” from a Registered Report journal.	Protocols of Registered Reports in the aging field should be registered using this template.
“Secondary Data Preregistration” (https://osf.io/x4gzt/)	This template is useful when registering a research project that uses an existing data set.	We encourage aging researchers to register studies involving secondary analyses of existing data using this format.

One frequently raised objection in the aging community is that much of our research in gerontology involves secondary analyses of existing data, and that makes preregistration inappropriate. Although preregistration for existing data may not be as intuitive as it is for an experimental study conducted in a laboratory, it is nonetheless quite important: HARKing (ie, hypothesizing after results are known) is a particular temptation, especially with large data sets where many combinations of variables may reach the .05 level of statistical significance. P-hacking in the form of fishing for significant results is a related temptation. Treating these (possibly spurious) findings as confirmatory rather than exploratory would make readers put greater weight on the robustness of these findings than the data observe. As discussed earlier, we believe that exploratory analyses should still be included in manuscripts, but labeled as such. Then, readers should treat them with less confidence compared with confirmatory findings. One possibility is replicating the findings in other data sets (or by reserving part of a data set for confirmatory analyses after the first-pass exploratory ones). Of course, this may not be possible for some topics or some data sets.

Happily, open science proponents, like those involved in SIPS (Society for the Improvement of Psychological Science), are creating preregistration templates that are appropriate for analyses of existing data sets, even ones where researchers have some experience with the variables already (such as new waves of longitudinal data, where the same variables have already been analyzed cross-sectionally). Weston and colleagues (https://osf.io/x4gzt/) are currently working on a template designed specifically for research using existing data that could be used by a range of disciplines (also included in Table 1). Here, researchers are asked a series of questions about their knowledge of the existing data set, prior work based on the data set (eg, what variables have been used before) and publications and presentations based on the data, before they are asked to elaborate on the new project. This is useful in demonstrating how much knowledge researchers had of the data before conducting the new, preregistered analyses, as well as showing that decisions to be made about the analysis have been done a priori rather than while also conducting the key analyses. Although this as well as other forms are not yet available as regular registration forms, it is possible to complete the template as a word doc or on a wiki and then register using one of the simple OSF forms such as the “OSF Standard Pre-Data Collection” form. Relatedly, the *Journals of Gerontology, Series B: Psychological Sciences* are now encouraging authors to submit articles for a special issue: Preregistered Studies of Personality Development and Aging Using Existing Data. The aim of this special issue was to highlight the benefits of preregistration in the context of existing data and how to overcome challenges in preregistration with existing data sets.

Another frequent concern among aging researchers is that open science practices, especially concerning sample sizes, will create the need for samples that are too large for some researchers to accomplish. This has been a topic of great discussion in areas of psychology that have similar concerns, but what seems agreed upon is that underpowered studies are equally uninformative regardless of the difficulty of recruiting participants. Given this, aging researchers will need to think more creatively about pursuing research designs that they have the resources to power (this may involve reducing individual differences variables considered beyond the age variable: https://approachingblog.wordpress.com/2018/01/24/powering-your-interaction-2/).

Another intriguing option for aging researchers concerned about having the resources to test an adequately-sized sample is to look for researchers willing to exchange samples. One exciting development is the Psychological Science Accelerator, a globally distributed network of more than 400 psychological science laboratories that coordinates data collection for selected studies (https://psysciacc.org/). Child development researchers have already started doing this in the Many Babies Project (Frank *et al.*, 2017). Although open science practices may complicate things for individual researchers and may make some projects more challenging to make the literature more credible, the good news is that colleagues are already working on solutions that may make projects more feasible even under newer expectations for sample sizes.

## Reflections: The (Open) Future of Gerontology

Implementing transparent open science practices will not be easy for gerontologists, as for any researchers. Many different entities must take responsibility and work together to make these changes possible.

From a top-down perspective, high-impact journals certainly do not discourage preregistration, and at least some journals actively encourage researchers to make their data sets and codes available upon request. However, editors may be hesitant to make these open science practices mandatory requirements to submit a manuscript, perhaps because they are concerned that this will stop some researchers from submitting articles to their journal. We encourage editors from the top aging journals to collaborate on shared policies that encourage open science practices.

Moreover, journals tend to prefer novelty and strong appeal of a manuscript while putting perhaps less value on the robustness of its scientific claims, a tendency that lowers transparency of the research study as well as its credibility (Giner-Sorolla, 2012; Vazire, 2017b). In the search for eye-catching results, journals may be tempting authors to use QRPs indirectly (eg, *p*-hacking, HARKing). Without high levels of transparency in scientific articles, it is difficult to detect the difference between weak and high-quality findings (Vazire, 2017a). Editors as well as reviewers therefore play a significant role in promoting transparency to increase readers’ accurate interpretation of research, and part of that may involve downplaying novelty in the review process, to increase credibility. *Nature* is a good example of a journal emphasizing transparency by providing more space for methods sections and creating a checklist of necessary technical and statistical information. We hope that other journals will follow this example even though some journals would have to extend the requirements for the manuscripts’ length.

Research grant funders like NIH can also influence open science practices positively because researchers are willing to follow their guidelines to obtain funding. Some grants already have certain open science requirements: NIH requires that articles are made available via self-archiving (“green OA”) and NSF requires researchers to submit a plan specifying data management and availability. Ideally, grant funders might be able to collaborate on establishing some standardized open science guidelines. For example, funders could implement rules as to what extent funded projects will mandate data and code availability for other researchers.

Universities are another central component in promoting open science. Open science may provide opportunities to broaden research findings with an eye towards education, such as the development of tutorials on new techniques. These approaches can then benefit the entire scientific discipline (Howe *et al.*, 2017). However, an essential challenge with open science involves time and money. It takes researchers time to format, annotate, and publish data as well as to learn new skills and software that allow automated analysis and documentation for others to use it. Educating people and incorporating infrastructural changes cost money, but how and who should cover these costs? (Howe *et al.*, 2017). Grants are predominantly interested in supporting expenses for direct research costs and it will be important to acknowledge open science as a fundamental budget item. Importantly, when researchers spend time on promoting and engaging in open science practices, it takes away time from writing articles and grant proposals. Because peer-reviewed publications are fundamental for a successful research career, institutions may think of ways to acknowledge and reward efforts in open science practices too (Howe *et al.*, 2017).

From researchers’ perspective, the issue is not simply a matter of changing our business practices in research, such as remembering to preregister before starting a study or making the raw and/or cleaned data publicly available. Some of these changes will require significant time and effort, such as ensuring that data are in a format where they easily can be made available to other researchers, while also maintaining participant confidentiality. Realistically, some open science practices may lead researchers to publish less frequently; changing expectations about sample sizes means that studies will take longer to conduct. Ultimately, this may lead researchers to publish less. Conversely, if use of preregistration and registered reports increases, along with a greater awareness of equivalence testing (Lakens *et al.*, 2018) that can provide quantitative support for potentially meaningful null age effects, leads to more published null age difference studies, this may provide a counterbalancing influence on publication rates.

Emphasizing better research transparency may also require changes in laboratory culture. Rouder, Haaf, and Snyder (2019) promote five different laboratory strategies that contribute to more transparent research (especially for quantitative research): (a) learning from mistakes as a team, (b) using computer automation in data and metadata collection when possible, (c) organizing research elements similarly by all lab members, (d) preferring coded rather than menu-driven analyses (e) implementing expanded documents that record how analyses were conducted. These changes will help to catch mistakes among lab members, and they will also help reviewers and other researchers who aim to replicate or use the material later on. Although incorporating and learning new routines can be time-consuming, and in some cases expensive, we believe this to be necessary and we encourage all laboratories to discuss ways to improve their practice and transparency in their everyday routines.

Although adopting open science practices has the potential to create challenges, we truly believe it is nonetheless vital for gerontologists to implement at least some of these practices. A transparent science of aging will contribute, together with more robust research practices, to a more credible science of aging. Ultimately, adopting open science practices will not only benefit aging researchers, but also the public who depends on our research findings.

## Funding

None reported

## Conflict of Interest

None reported.
